# Adaptive Response of *Listeria monocytogenes* to the Stress Factors in the Food Processing Environment

**DOI:** 10.3389/fmicb.2021.710085

**Published:** 2021-08-19

**Authors:** Natalia Wiktorczyk-Kapischke, Krzysztof Skowron, Katarzyna Grudlewska-Buda, Ewa Wałecka-Zacharska, Jakub Korkus, Eugenia Gospodarek-Komkowska

**Affiliations:** ^1^Department of Microbiology, L. Rydygier Collegium Medicum in Bydgoszcz, Nicolaus Copernicus University in Toruń, Bydgoszcz, Poland; ^2^Department of Food Hygiene and Consumer Health, Wrocław University of Environmental and Life Sciences, Wrocław, Poland

**Keywords:** *Listeria monocytogenes*, stress factors, adaptive response, disinfectants, food processing environment

## Abstract

*Listeria monocytogenes* are Gram-positive, facultatively anaerobic, non-spore-forming bacteria that easily adapt to changing environmental conditions. The ability to grow at a wide range of temperatures, pH, and salinity determines the presence of the pathogen in water, sewage, soil, decaying vegetation, and animal feed. *L. monocytogenes* is an etiological factor of listeriosis, especially dangerous for the elderly, pregnant women, and newborns. The major source of *L. monocytogenes* for humans is food, including fresh and smoked products. Its high prevalence in food is associated with bacterial adaptation to the food processing environment (FPE). Since the number of listeriosis cases has been progressively increasing an efficient eradication of the pathogen from the FPE is crucial. Understanding the mechanisms of bacterial adaptation to environmental stress will significantly contribute to developing novel, effective methods of controlling *L. monocytogenes* in the food industry.

## Introduction

*Listeria monocytogenes* are Gram-positive, rod-shaped, non-spore-forming bacteria, widespread in the environment, i.e., water, soil, sewage, decaying vegetation, silage, and animals (O'Neil and Marquis, 2006; Gandhi and Chikindas, 2007). These bacteria tolerate low water activity (≥ 0.9) and are able to survive in a wide range of temperature (0–45°C), pH (4.3–9.6), and salinity (up to 10% NaCl; Renier et al., 2011). *L. monocytogenes* is a causative agent of human listeriosis, especially dangerous for the elderly, pregnant women, newborns, and immunocompromised patients. The major source of the pathogen for humans is food, including meat, fish (raw and smoked), mold cheese, unpasteurized milk, ready-to-eat (RTE) food, and fresh produce (Gandhi and Chikindas, 2007). Despite the low annual incidence of listeriosis ranging from 0.1 to 10 cases per million people, the mortality rate is high and may exceed 15–20% (World Health Organization (WHO), 2018a). To date, many listeriosis outbreaks have been noted worldwide, including the deadliest outbreak in United States history (1985 – soft cheese; Beckers et al., 1987), the deadliest outbreak in Canada (2008 – deli meat; Birk-Urovitz, 2011), and the deadliest outbreak ever in South Africa (2017–2018, deli meat; Smith et al., 2019). Table 1 summarizes data on the most important epidemics of listeriosis. Despite the development of therapies and biocontrol measures, *L. monocytogenes* remains a serious threat to food safety, as evidenced by the outbreak in South Africa. Environmental stress affects the morphology, pathogenicity, gene expression, and antimicrobial resistance (AMR) of *L. monocytogenes* (Matereke and Okoh, 2020). Understanding the adaptive mechanisms of the pathogen can facilitate the planning of disinfection strategies (more effective and less expensive methods) in a food processing environment (FPE), thus ensuring food safety.

**Table 1:** The most important epidemics of listeriosis.

	Year	Country	Food	Number of cases	Number of deaths	Number of miscarriages	References
United States	1985	Los Angeles	Mexican-style cheese	142	41	0	Beckers et al., 1987
	1998–1999	11 states	Hot dog	108	14	4	Centers of Disease Control and Prevention (CDC), 1999
	2000	10 states	Deli turkey meat	29	4	3	Centers of Disease Control and Prevention (CDC), 2000
	2002	Eight states	Fresh and frozen, Ready-to-eat turkey, and chicken	46	7	3	Centers of Disease Control and Prevention (CDC), 2002
	2010	Texas	Pre-cut celery	10	5	0	Gaul et al., 2012
	2011	28 states	Cantaloupe	147	33	1	Centers of Disease Control and Prevention (CDC), 2012a
	2012	13 states	Ricotta salata Cheese	22	4	1	Centers of Disease Control and Prevention (CDC), 2012b
	2013	Five states	Crave brothers farmstead cheeses	6	1	1	Centers of Disease Control and Prevention (CDC), 2012c
	2010–2015	Four states	Blue bell ice cream	10	3	0	Centers of Disease Control and Prevention (CDC), 2015a
	2015	10 states	Soft cheeses distributed by Karoun dairies	30	3	1	Centers of Disease Control and Prevention (CDC), 2015b
	2014–2015	12 states	Caramel apples	35	7	1	Centers of Disease Control and Prevention (CDC), 2015c
	2016	Two states	Raw milk	2	1	0	Centers of Disease Control and Prevention (CDC), 2016a
	2016	Four states	Frozen vegetables	9	3	0	Centers of Disease Control and Prevention (CDC), 2016b
	2016	Nine states	Packaged salads	19	1	0	Centers of Disease Control and Prevention (CDC), 2016c
	2017	Four states	Soft raw milk cheese Made by vulto creamery	8	2	0	Centers of Disease Control and Prevention (CDC), 2017
	2018	Two states	Deli ham	4	1	0	Centers of Disease Control and Prevention (CDC), 2018
	2019	Five states	Deli-sliced meats and cheeses	10	1	0	Centers of Disease Control and Prevention (CDC), 2019a
	2019	Four states	Pork products	4	0	0	Centers of Disease Control and Prevention (CDC), 2019b
	2019	Five states	Hard-boiled eggs	8	1	0	Centers of Disease Control and Prevention (CDC), 2020a
	2020	17 states	Enoki mushrooms	36	4	2	Centers of Disease Control and Prevention (CDC), 2020b
	2020	Four states	Deli meats	12	1	0	Centers of Disease Control and Prevention (CDC), 2021a
	2021	Four states	Queso Fresco	11	1	0	Centers of Disease Control and Prevention (CDC), 2021b
Europe	2003	The Swindon area of the United Kingdom	Prepacked sandwiches from a retail outlet	5	0	0	Dawson et al., 2006
	2005	Switzerland	Soft cheese (known as “tomme”)	10	3	2	Bille et al., 2006
	2015–2018	Austria, Denmark, Finland, Sweden, and the United Kingdom	Frozen corn	41	6	0	European Centre for Disease Prevention and Control (ECDC), 2018a
	2017–2019	Netherlands, Belgium	Ready-to-eat meat products	21	3	1	European Centre for Disease Prevention and Control (ECDC), 2019a
	Sience 2015	Denmark, Germany, and France	Ready-to-eat salmon products	12	4	0	European Centre for Disease Prevention and Control (ECDC), 2018b
	2014–2019	Denmark, Estonia, Finland, France, and Sweden	Cold-smoked fish products	22	5	0	European Centre for Disease Prevention and Control (ECDC), 2019b
	2019	Spain	Chilled roasted pork meat product	222	3	6	World Health Organization (WHO), 2018b
Australia	2018	Australia	Rockmelons	20	7	1	World Health Organization (WHO), 2018c
Africa	2017–2018	Republic of South Africa	Ready-to-eat processed meat products	1,024	200	–	Smith et al., 2019

The review aims to summarize knowledge about the stress response of *L. monocytogenes* in FPE. A deeper insight into the stress response of *L. monocytogenes* may help implement well-designed food preservation and disinfection protocols, reducing the risk of the bacteria in the food and FPE and ensuring consumer safety.

## Stress Factors in the Food Processing Environment

**Figure 1:**
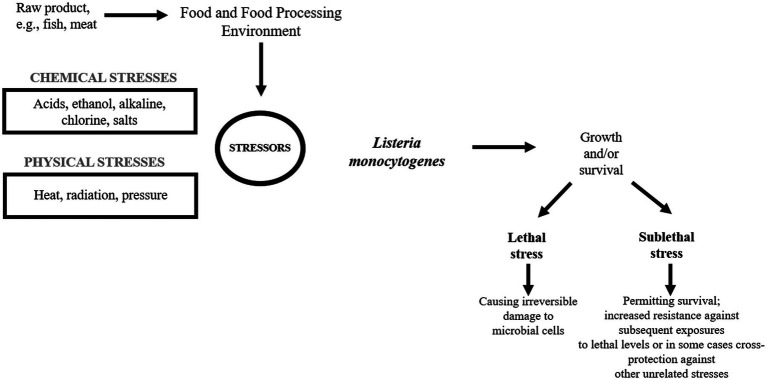
*Listeria monocytogenes* in the food processing environment.

*Listeria monocytogenes* is a serious problem in the food industry as the pathogen easily contaminates a variety of food products leading to food recall (Miladi et al., 2017). In FPE, *L. monocytogenes* encounters many adverse stress factors, e.g., low/high temperature, low pH, high salinity, and the concentration of chemicals, which may be sublethal or lethal (Figure 1).

**Figure 2:**
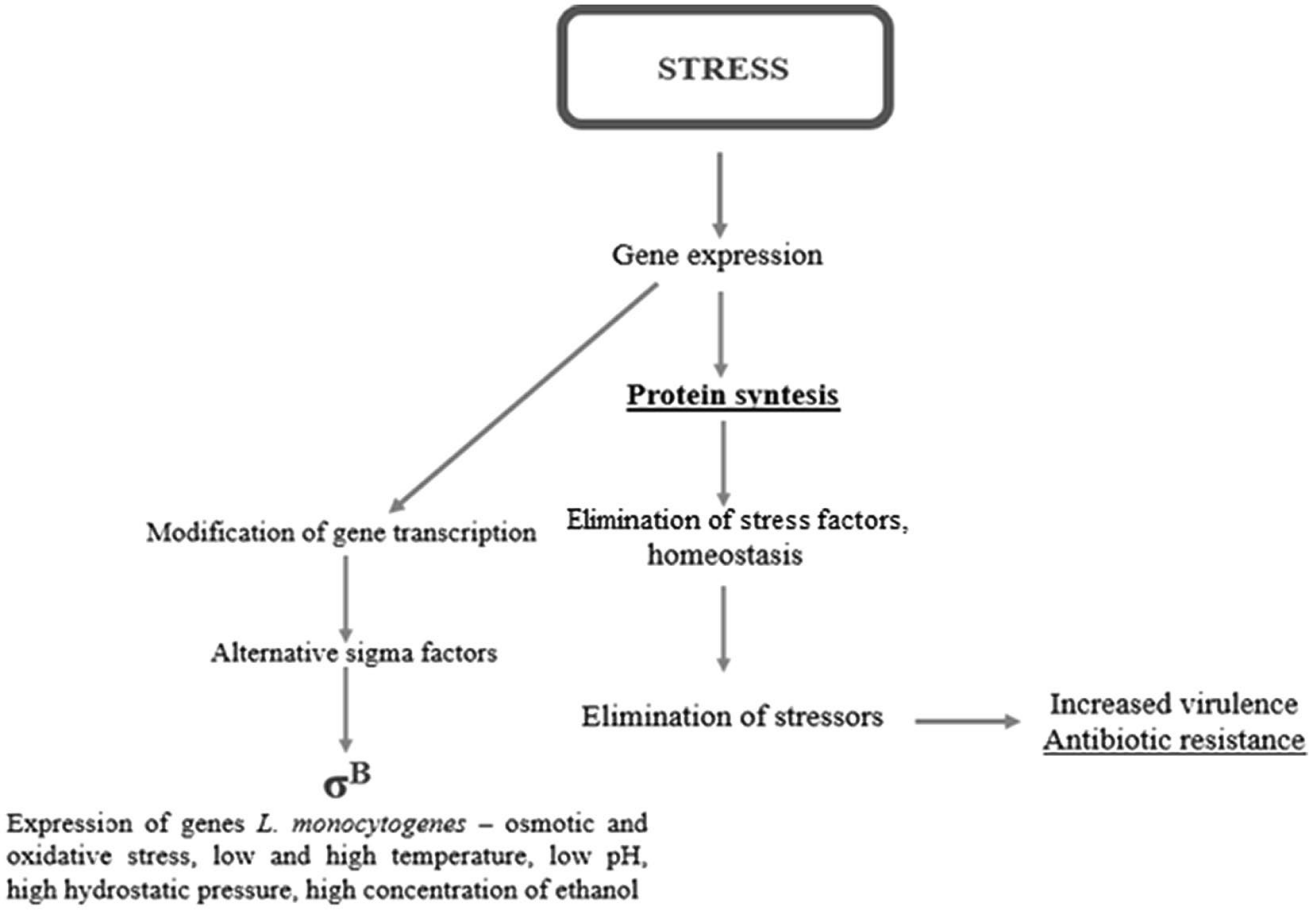
The stress response in *L. monocytogenes*.

Environmental stress tolerance and virulence may be considered as overlapping aspects of the biology of *L. monocytogenes* (O’Byrne and Karatzas, 2008). Without a strong stress response, the pathogen would not survive and persist in the FPE and withstand the passage through the human gastrointestinal tract (NicAogain and O’Byrne, 2016). One of the major stress response mechanisms is associated with alternative sigma factors σ^B^, σ^C^, σ^H^, and σ^L^, of which σ^B^ plays the pivotal role (Lungu et al., 2009). In *L. monocytogenes*, these factors control over 300 genes, including stress-associated and virulence genes. σ^B^ helps *L. monocytogenes* survive under acid, osmotic, oxidative, and other stress conditions (Figure 2). The activity of σ^B^ and hence the general stress response activation in *L. monocytogenes*, is regulated by a complex structure known as the stressosome (NicAogain and O’Byrne, 2016). Analyses of the proteome and transcriptome of *L. monocytogenes* revealed many proteins activated in response to stress. Table 2 presents an overview of *L. monocytogenes* main stress-response and virulence genes and proteins.

**Table 2:** *Listeria monocytogenes* stress-response and virulence genes and proteins.

Stress	System	Gene	Protein
Acid	Glutamate decarboxylase system (GAD)	*gadA* (formerly *gadB*, *gadD*, *gadD3*, or *lmo2434*)	Glutamate decarboxylase
		*gadCB* (formerly *gadD2T2* or *lmo2363–lmo2362*)	Glutamate decarboxylase and a glutamate-γ-aminobutyric acid (GABA) antiporter
Osmotic	Glycine betaine transport system	*betL*	Glycine betaine transporter (BetL)
	Glycine betaine ABC transport system	three-gene operon (*gbuABC*)	Gbu
	Carnitine ATP-binding cassette (ABC) transport system	Four-gene operon (*opuCABCD*)	OpuC
Cold	–	*cspD*	Cold-shock protein D
		*ltrC*	Low-temperature requirement C protein
Heat	–	*grpE*	Heat-shock protein GrpE
Oxidative	–	*lmo0669*	Oxidoreductase
		*lmo1433*	Glutathione reductase
Other genes involved in the stress response	–	*clpB*	ClpB, similar to endopeptidase Clp ATP-binding chain B; transcribed in an operon with *lmo2205*
	–	*clpP*	ClpP, an ATP-dependent Clp protease proteolytic subunit
	–	*clpX*	ClpX, an ATP-dependent Clp protease ATP-binding subunit
	–	*lmo1138*	Similar to ATP-dependent Clp protease proteolytic component
	–	*lmo1601*	General-stress protein; transcribed in an operon with *lmo1602*, which encodes an unknown protein
	–	*lstR*	Lineage-specific thermal regulator; transcribed in an operon with *sigC*
	–	*rsbVWX*	Regulators of σ^B^ activity, RsbV, RsbW, and RsbX
	Bile-exclusion system	*bilE* (formerly *lmo1421-lmo1422*, or *opuBAB*)	Two-gene operon (*bilEAB*) encodes a bile-exclusion system; responsible for bile resistance
	Bile tolerance	*bsh*	Bile salt hydrolase
	clpC operon	comprises *ctsR*, *mcsA*, *mcsB*, and clpC,	CtsR, McsA, and McsB, which both regulate CtsR activity, and the ClpC endopeptidase and chaperone

σ^B^ is responsible for the transcription of genes involved in the stress response. In turn, PrfA (transcriptional activator of virulence genes) controls the expression of virulence genes. There is an extensive cross-talk system between σ^B^ and PrfA ensuring the optimal expression of genes required in extrahost environments (including suppression of genes that reduce efficiency) and in intrahost environments (Gaballa et al., 2019). The stress conditions encountered during food production and the adaptive response of *L. monocytogenes* affect the virulence of the bacilli and thus pose a much higher risk to consumers. The knowledge on the adaptive mechanisms of *L. monocytogenes* to the changing environmental conditions will help design appropriate methods of production process control and disinfection (mainly lethal effect). As a result, we expect to limit the acquisition of resistance and prevent the increase of virulence of *L. monocytogenes*.

The presented review focuses on the stress factors encountered by *L. monocytogenes* in FPE and the stress response mechanisms.

### Salt

One of the main preservatives in FPE is salt contributing to osmotic stress. Salt is frequently used in the food industry as a preservative and antibacterial agent, especially in RTE meat and fish products (Desmond, 2006). In addition, salt is widely used as a texture and flavor improver (Ruusunen and Puolanne, 2005). Salt disturbs homeostasis between the internal and external environment of the bacterial cells. All changes of the osmolarity induce osmotic stress resulting in hypotonia (swelling of the cell) or hypertonia (shrinking of the cell; Sleator and Hill, 2002). The change of osmolarity may also affect biophysical properties (surface tension), decrease cell membrane fluidity, and destroy proteins and DNA. Moreover, salt can contribute to bacterial cell desiccation, making it vulnerable to the action of free radicals, which results in cell death. Reversely, osmotic stress may lead to cross-resistance and help bacteria survive in the next stage of food processing (Burgess et al., 2016).

**Figure 3:**
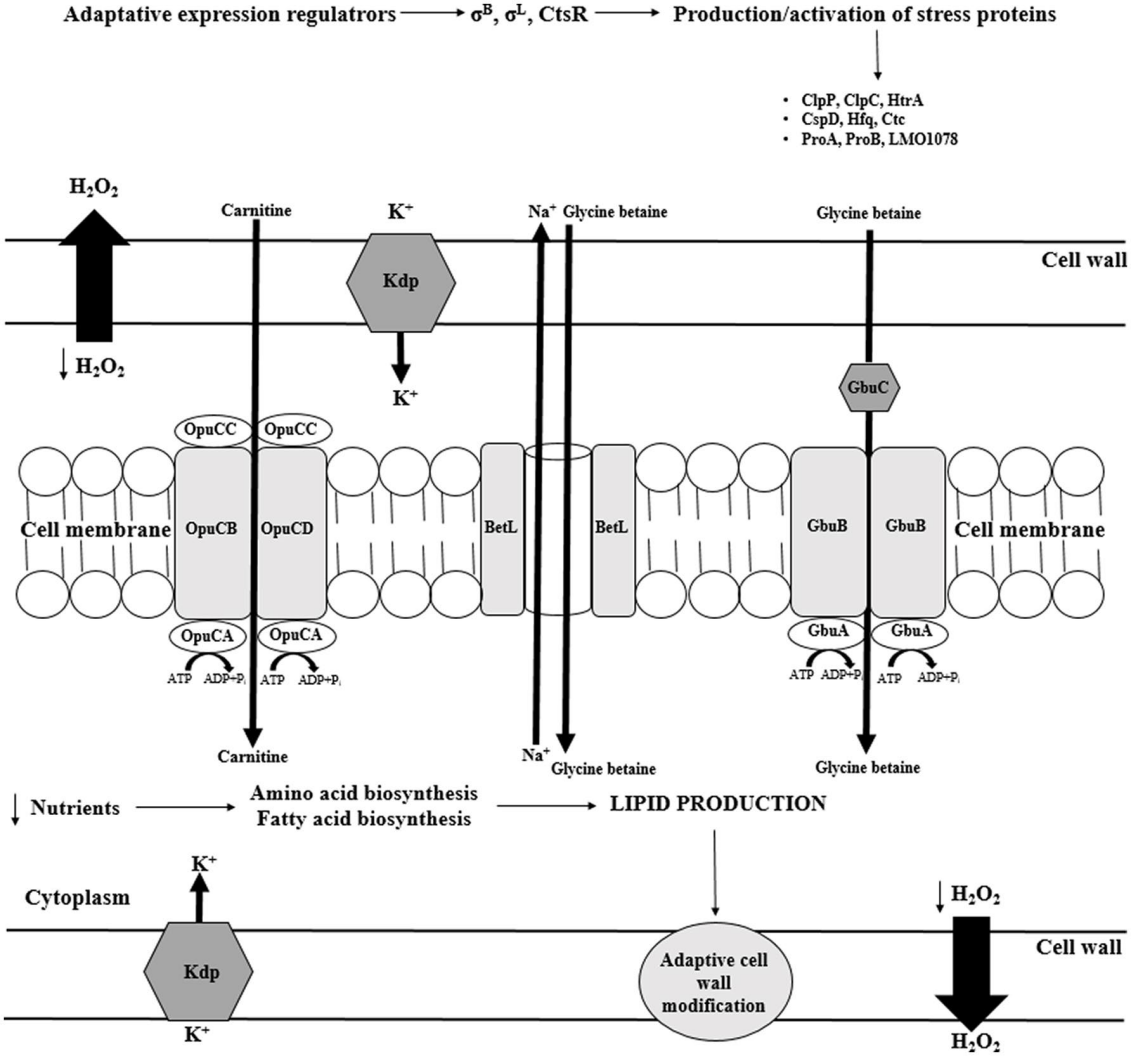
Transport of compatible solutes in response to osmotic stress in *L. monocytogenes* (CtsR – negative transcription regulator; ClpP, ClpC – ATP-dependent *Clp* protease; HtrA – serine protease; Csp – cold shock proteins; Hfq – the RNA binding protein; Ctc – 50S ribosomal protein L25; ProBA – proline synthesis enzyme system; Kdp – operon response regulator; Gbu – glycine betaine; OpuC – ATP-dependent carnitine transporter; ATP – adenosine triphosphate; ADP – Adenosine 5'-diphosphate; and BetL – glycine betaine transporter).

*Listeria monocytogenes* can withstand up to 10% of NaCl in the environment (Chihib et al., 2003). Many proteins play role in salt stress response, including osmolyte transporters, cell wall modifying proteins, regulatory proteins, and general stress response proteins (Figure 3). The response to increased osmolality in *L. monocytogenes* is two-step. First, K^+^ and glutamate are collected, and then the accumulation of compatible solutes/osmolytes occurs. Compatible solutes are small organic molecules that function as osmoprotectants and are indispensable for cell turgor maintenance. In response to elevated osmolarity, bacterial cell accumulates very high levels of compatible solutes (restoring the cell turgor without the influence on the cytoplasmic functions). Additionally, compatible solutes stabilize the structure and functions of enzymes under stress conditions (Matereke and Okoh, 2020). A number of osmolytes have been identified in *L. monocytogenes*, e.g., glycine betaine, carnitine, proline, acetylcarnitine, gammabutyrobetaine, and dimethylsulfoniopropionate. Since these bacteria are unable to synthesize osmolytes *de novo* they must be transported from the environment. L-carnitine is commonly found in raw meat, whereas vegetables (sugar beet and spinach) and cornflakes are sources of glycine betaine (Chan et al., 2007).

The analysis of *L. monocytogenes* genome revealed three σ^B^–dependent transporters of compatible solutes, i.e., betaine symporter (BetL), Gbu, and OpuC (Figure 3; Bucur et al., 2018). Glycine betaine accumulation in the cytosol may occur without impairing protein structure and enzyme-substrate interaction (Angelidis and Smith, 2003). There are two transporters of glycine betaine, i.e., ATP-dependent porter II (Gbu) and Na^+^- glycine BetL. BetL, encoded by *betL* gene, is activated immediately upon stress exposure but provides long-term protection only under low salt stress. In turn, Gbu, encoded by *gbuABC* operon, plays the predominant role in long-term adaptation, especially at higher concentrations of salt (Mendum and Smith, 2002). OpuC, encoded by *opuCABCD* operon, is an ATP-dependent carnitine transporter activated in response to osmotic and cold stress (Figure 3; Bucur et al., 2018). In the absence of osmoprotectants, the osmotic stress response mechanism stress relies on Ctc protein. Ctc most likely binds to the 5S RNA sub-principle (Duché et al., 2002).

An important role in osmotolerance plays also general stress response proteins, including serine protease HtrA (degradation of improperly folded proteins), two-component regulatory system LisRK, ClpC (ATPase), CLpP (protease), and RelA (synthesis of guanosine pentaphosphate [(p)ppGpp]), Ctc. Researchers have suggested that RelA is involved in osmotic stress response independent of compatible solutes accumulation (Burall et al., 2012).

In σ^B^ regulon, scientists have described eight genes encoding putative osmotic stress-associated proteins, i.e., *LMRG 01658*, *LMRG 00208*, *LMRG 00211-00212*, *hfq*, and *dtpT* in *L. monocytogenes*. However, the role of these genes has not been elucidated yet (Liu et al., 2019). Ribeiro et al. (2014) have suggested that under salt stress, upregulation of these genes enables the increased synthesis of exopolysaccharides. The Hfq protein binds sRNA during intracellular growth. The mutants devoid of this protein were not able to respond properly to osmotic and ethanol stress (Christiansen et al., 2006). The DtpT protein plays important role in the transport of di- and tripeptides involved in osmotic stress protection (Wouters et al., 2005). In turn, Kdp is a transcriptional response regulator enabling quick reaction to stress *via* K^+^ import. Also, trehalose was reported to be linked to osmotolerance, and the *treA* gene, encoding phosphotrehalase was detected in *L. monocytogenes* (Ells and Truelstrup Hansen, 2011). Researchers described proteins modifying cell membrane, i.e., putative peptidoglycan-associated protein (*lmo2085* gene) and putative UDP-glucose phosphorylase (*lmo1078* gene) as osmotic stress proteins (Burgess et al., 2016).

The main mechanism of the osmotic stress response in *L. monocytogenes* involves osmoprotectants. In their absence, Ctc protein provides protection. The contribution of the remaining proteins presented is not fully understood. Food manufacturers should pay attention to the rightness of using salt as a preservative in the elimination of microorganisms from food, especially *L. monocytogenes*, due to the advanced mechanisms of adaptation to high salt concentration.

In recent years, there has been a demand for low-salt products, which forces searching for new technologies to control microbiological hazards in low-salt products. The currently used alternative to salt (especially in meat products) is the treatment with high isostatic pressures, activated plasma, pulsed UV light, or active packaging (Fraqueza et al., 2021). In our opinion, an attractive solution would be natural substances with antimicrobial activity (e.g., essential oils and plant extracts).

### Temperature

Another factor ensuring food safety is temperature, both high and low. Heat treatment (pasteurization and sterilization) is one of the most common methods of assuring food safety and microbial elimination. However, too low temperature of such a process might not effectively eliminate the pathogen, and too high temperature may affect the organoleptic properties of food. In turn, low temperatures (e.g., cold stores) aim to limit the growth of undesirable microorganisms in food.

*Listeria monocytogenes* is able to survive and grow in a wide temperature range, i.e., 0–45°C (Arioli et al., 2019). Depending on the physiological state of the cell, inactivation of *L. monocytogenes* ranges from 10 min to 12 s at 55–65°C (Smelt and Brul, 2014).

**Figure 4:**
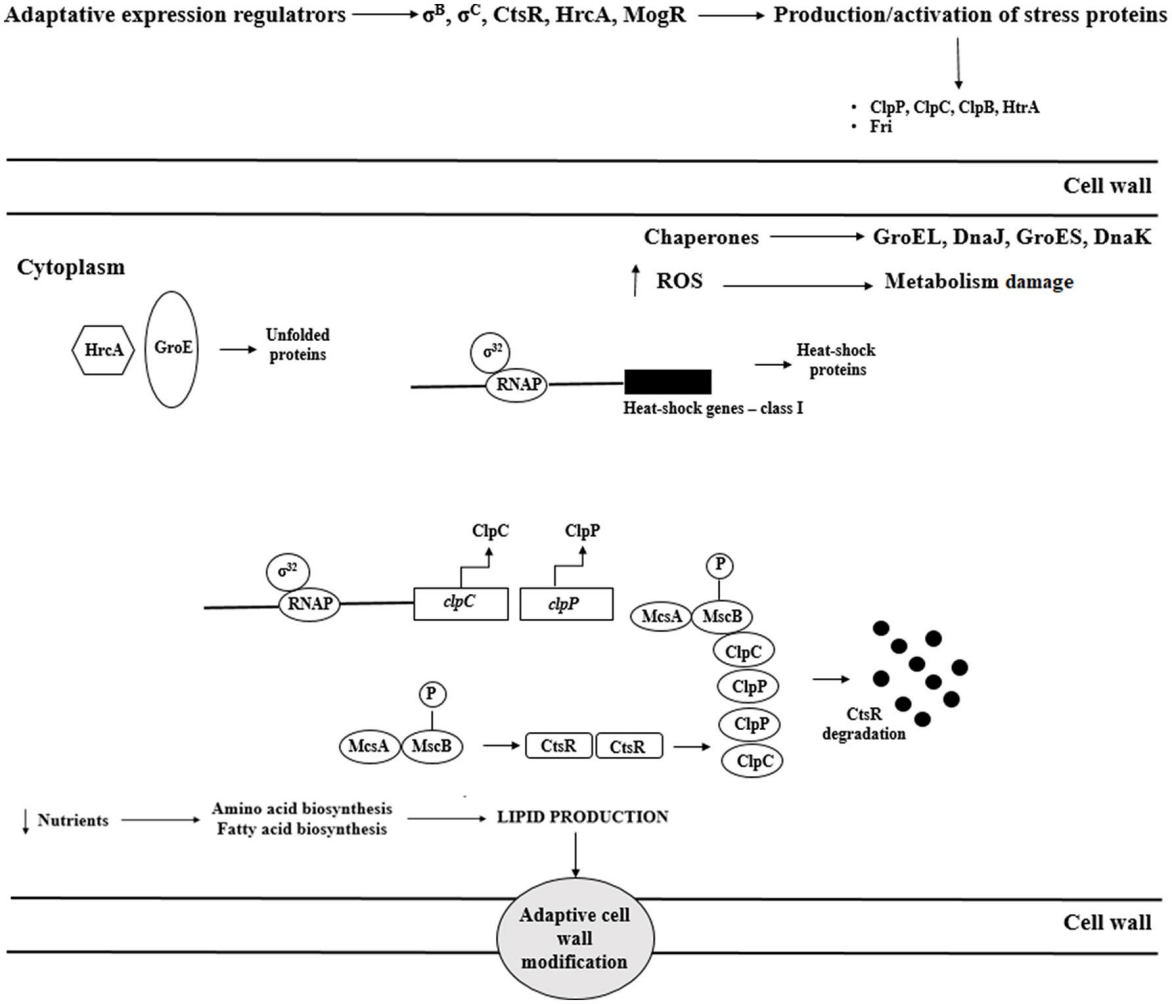
Regulation of heat shock response of *L. monocytogenes* (ROS – reactive oxygen species; Msc – tyrosine kinase; McsA – zinger finger protein; HtrA – serine protease; ClpC – ATP-dependent *Clp* protease; HrcA – Heat-inducible transcription repressor; CtsR – negative transcription regulator; Fri – ferritin-like protein; and RNAP – RNA polymerase enzyme).

Reaction to elevated temperature is associated with increased expression of heat shock proteins (Hsp). Hsp stabilize proteins and prevent improper folding and aggregation of proteins (Figure 4). In response to heat stress, *L. monocytogenes* increase the production of these proteins, thereby stimulating the repair of thermally denatured proteins. *Listeria monocytogenes* possess III classes of heat shock-associated genes. Genes of class I (*groE*, *dnaK*, *dnaJ*, *groEL*, and *groES*) are overexpressed during the accumulation of denatured proteins in the cytosol and act as intracellular chaperones. Class III genes encode ATP-dependent proteins of caseinolytic activity (ClpB, ClpC, ClpP, and ClpE; Bucur et al., 2018). These proteins cooperate to maintain the proper protein structure at high temperatures. The proteins GroEL and GroES (regulation of basic cellular processes) and DnaK, and DnaJ (stabilization of the conformation of unfolded proteins) are the main chaperones protecting *L. monocytogenes* against heat stress. Also, ClpE and ClpC (caseinolytic activity) are indispensable during the replication at elevated temperatures and nutrients deficiency. During heat stress, these proteins participate in the regulation of proteolysis and proper protein folding. Genes of class I and III are negatively regulated by HrcA and CtsR, respectively, whereas class II genes are under positive control of σ^B^ (Nair et al., 2000). GroE regulates HrcA activity. Under optimal growth conditions, the interaction of HrcA with GroE inhibits transcription of class I genes. At elevated temperature, unfolded proteins bind GroE contributing to HrcA inactivation and enabling binding of RNA-s32 polymerase with promoters and gene expression.

At 37°C, CtsR is stabilized by McsA, which results in class III gene repression. At increased temperature, McsB modifies CtsR conformation preventing its binding with gene promoters. As a result, RNA-s32 polymerase binds with promoters leading to gene expression, and CtsR is degraded (Figure 4; Kocaman and Sarimehmetoğlu, 2016). ClpC expression is negatively controlled at the transcription level, either directly or indirectly, by PrfA (Ripio et al., 1998).

**Figure 5:**
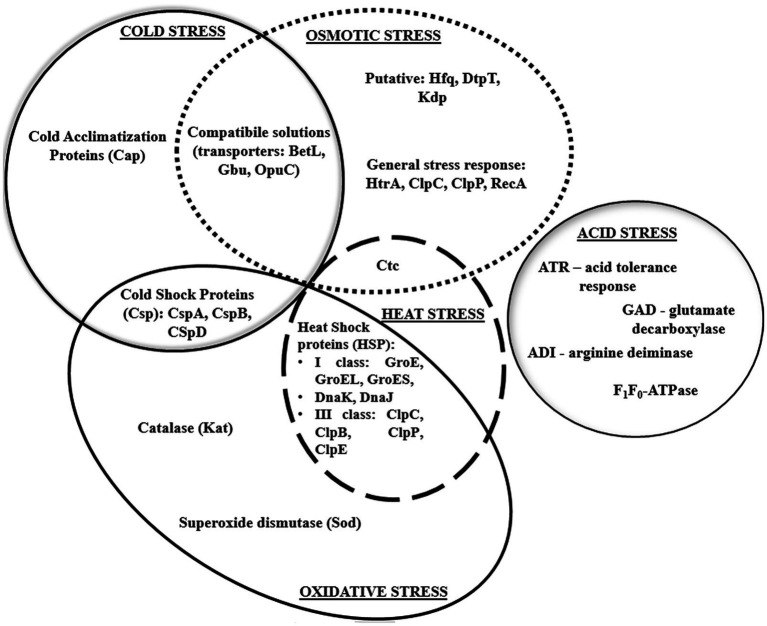
Interaction of various stress response mechanisms in *L. monocytogenes*.

Heat shock proteins, i.e., DnaK, ClpC ATPase, ClpP serine prosthesis, and Ctc protein also participate in the osmotic stress response. Thus, proteins involved in the reaction to osmotic and heat stress interact with each other (Figure 5).

**Figure 6:**
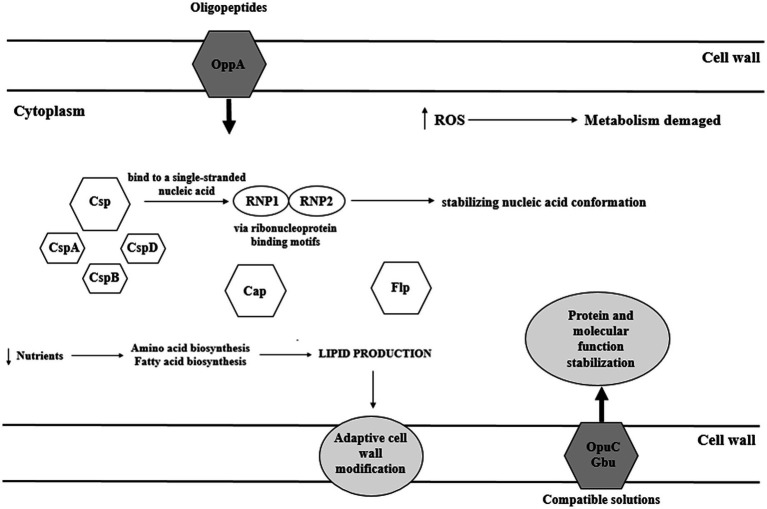
Cold stress response of *L. monocytogenes* (OpuC – ATP-dependent carnitine transporter; Gbu – glycine betaine; ROS – reactive oxygen species; Csp – cold-shock protein; Cap – cold acclimatization protein; and Flp – ferritin-like proteins).

During food processing, microbes experience low temperatures. Cold stress response in bacteria is three-step, i.e., cold shock, acclimatization, and adaptation (Kocaman and Sarimehmetoğlu, 2016). At 10°C, amino-acid starvation, oxidative stress, synthesis of misfolded proteins, reconstruction of a cell wall, changes of metabolism, and induction of global regulatory reactions occur (Lianou and Sofos, 2007). A fast decrease below optimal temperature, termed cold shock, triggers the synthesis of cold shock proteins (Csp). Csp are small proteins (65–70 aa) of highly conserved structure, expressed at a low level at optimal temperature (37°C; Schmid et al., 2009). Csp function as a molecular chaperone, enabling replication, transcription, and translation at low temperatures (Lee et al., 2012). Csps bind to a single-stranded nucleic acid *via* ribonucleoprotein binding motifs RNP1 and RNP2, thereby stabilizing nucleic acid conformation and preventing its degradation (Barria et al., 2013). CspA, CspB, and CspD contribute to adaptation to a varying extent (Schmid et al., 2009). Schmid et al. (2009) have shown that *L. monocytogenes* ∆cspA and ∆cspD mutants significantly worse responded to cold stress. Stress conditions also induce cold acclimatization proteins (Cap; Figure 6; Barria et al., 2013). The main mechanism of response to cold stress in *L. monocytogenes* relies on the action of both, Csp and Cap proteins. There are also other mechanisms involved in the cold stress response, supporting Csp and Cap action. Another cold stress response mechanism involves osmolytes and cryoprotectants (Angelidis and Smith, 2003). A key role as a cryoprotectant is attributed to branched-chain fatty acids of the cell membrane, ensuring membrane integrity at low temperatures (Becker et al., 2000). During cold shock, σ^B^ confers growth phase-dependent adaptation and assures efficient accumulation of cryoprotectants such as betaine and carnitine. Eight Rsb proteins modulate σ^B^ binding to the primary regulator through protein-protein interaction and phosphates transfer (Liu et al., 2019).

High temperature is a frequently used factor for the elimination of microorganisms in the food industry. To prevent loss of organoleptic properties, producers shorten the thermal processing of food. Unfortunately, such an approach may generate sub-lethal stress leading to the resistance acquisition in microorganisms. *Listeria monocytogenes* has developed several adaptation mechanisms to both high and low temperatures. Therefore, food producers should consider combining the thermal treatment with other methods, e.g., chemicals or natural preservatives (e.g., bacteriocins). The combination of methods can provide a lethal effect on microorganisms (safety for the consumer) without affecting the properties of the food.

### pH

Acid stress is one of the most common stresses faced by foodborne pathogens. Low pH increases H^+^ concentration, decreasing an internal pH (pHi) and inhibiting microbial growth. The ability to withstand acid stress determines bacterial survival in acid food products (Ryan et al., 2008). Bacteria need to restore homeostasis inside the cell *via* a passive mechanism (an increase of cytoplasm buffer capacity) or an active one (using a proton pump) to survive (Ryan et al., 2008). In food processing plants, *L. monocytogenes* is exposed to many acids (benzoic, salicylic, lactic, and propionic acid), commonly used as food preservatives and disinfectants. Other acid challenges, i.e., gastric juice and bile salts, the pathogen encounters in the human food tract (Begley et al., 2002).

**Figure 7:**
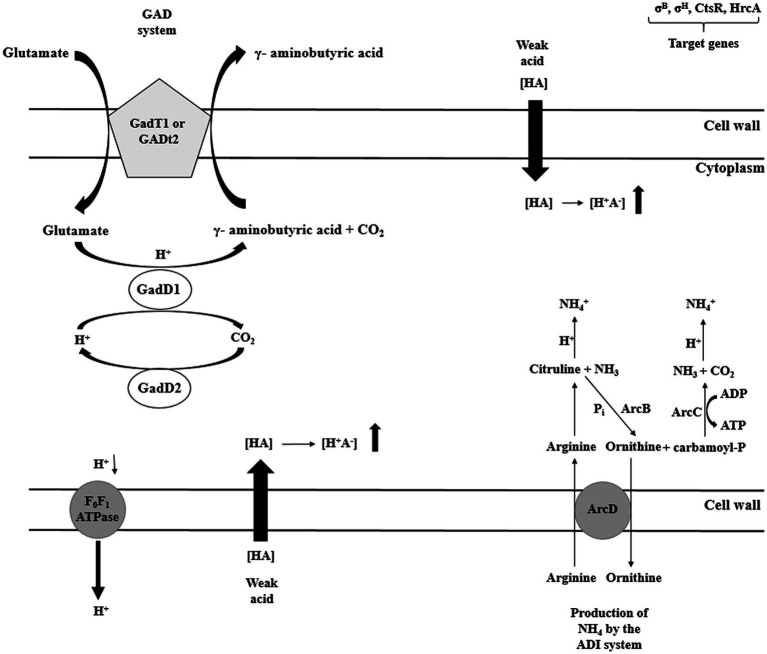
Selected mechanisms of acid stress response of *L. monocytogenes* (GAD – glutamate decarboxylase system; ADI – arginine deiminase system; HrcA – Heat-inducible transcription repressor; CtsR – negative transcription regulator; ArcA – arginine deiminase; ArcB – catabolic ornithine carbamoyltransferase; ArcC – carbamate kinase; ArcD – membrane-bound arginine-ornithine antiporter; and F_0_F_1_ –ATPase – proton efflux membrane ATPase).

There are four potential mechanisms responsible for the homeostasis maintenance in *L. monocytogenes*, i.e., acid tolerance response (ATR), glutamate decarboxylase (GAD), arginine deiminase (ADI), and F_1_F_0_-ATPase (Cotter et al., 2005). The major role of ATR mechanism is the cell protection against the lethal effect of stress following short exposure to mild acids (Koutsoumanis et al., 2003). The GAD system affects survival in food products of low pH (juices, yogurts, salads, and mayonnaise). The GAD system compromises five genes. Three genes (*gadD1*, *gadD2*, and *gadD3*) encode decarboxylases and two genes encode (*gadT1*, *gadT2*) antiporters (Melo et al., 2015). The GAD system converts extracellular glutamate to γ-aminobutyrate acid (GABA), elevating pHi (Werbrouck et al., 2009). Subsequently, GABA is exchanged for glutamate at the cell membrane by GadT2 antiporter. As a consequence, an intracellular proton is consumed, contributing to alkalization of the environment and pH homeostasis. Researchers have shown that strains with low GAD activity are susceptible to gastric juice (Ryan et al., 2009). Extremely acid pH activates another system: the ADI pathway (Soares and Knuckley, 2016). ADI converts, imported from the external environment, arginine to ornithine, CO_2_, ammonia, and ATP. Three enzymes participate in the arginine conversion, i.e., ADI, carbamoyltransferase, and carbamate kinase, encoded by *arcA*, *arcB*, and *arcC*, respectively. Ornithine is then transported from the cell, in an energy-independent manner, *via* membrane-bound antiporter, encoded by *arcD* gene. In turn, ammonia, produced as a by-product, reacts with intracellular protons generating NH4^+^, thereby increasing cytoplasmic pH and protecting the cell from the acid environment (Figure 7; Matereke and Okoh, 2020). ATP produced during arginine conversion may be used by F_1_F_0_-ATPase. This multisubunit enzyme generates a proton gradient, enabling H^+^ expulsion and homeostasis restoration (Ryan et al., 2009). The enzyme consists of two distinct domains: the membrane domain F_0_, which functions as a channel for protons translocation, and the cytoplasmic domain F_1_ catalyzing ATP synthesis and hydrolysis (Figure 7; Smith et al., 2013). The three discussed systems, i.e., ATR, GAD, and F_1_F_0_-ATPase, act at the same time (Figure 5). Interactions between them ensure survival and adaptation to acid stress.

In the food production environment, *L. monocytogenes* may also face sublethal alkaline stress associated with the use of detergents and disinfectants. The adaptation to such conditions involves increased production of acids and induction of transporters and enzymes responsible for protons retention and cell surface modifications (Soni et al., 2011). Usually, in response to high pH, the general stress proteins production, ATP generation (*via* ATP synthase conjugation with H^+^), and increased expression and activity of the monovalent cation/proton antiporters are observed (Giotis et al., 2010).

Many foodborne pathogens, including *L. monocytogenes*, develop adaptation to low pH and cross-resistance to other stresses upon exposure to acid stress. This cross-protection phenomenon may have severe consequences for food safety, as *L. monocytogenes* can survive various treatments, such as heat stress, cold stress, and osmotic stress. It is necessary to search for new disinfection methods in the food industry, limiting acids and alkalis use. An attractive solution could be substances of natural origin (herbs extracts and essential oils) or bacteriocins (environmentally friendly and safe for the consumer). Lately, increasing attention is being paid to essential oils as natural additives to extend the shelf life of food products (Tongnuanchan and Benjakul, 2014). Work (Shan et al., 2009; Efenberger-Szmechtyk et al., 2021) on natural preservatives is ongoing, and the results are promising. It is also necessary to understand the impact of such new applications on the survival of *L. monocytogenes*.

### Nutrients

The availability of ingredients in the FPE varies. Availability is low in post-disinfection areas. In turn, during food processing, there may be different types of waste, such as blood, fruit juices, etc. The availability of nutrients influences microbial biofilm formation, undesirable in the food industry. *Listeria monocytogenes* is able to use various compounds in the form of carbon, and/or energy sources to thrive in potential niches also within the food industry (Tapia et al., 2020). Thus, *L. monocytogenes* can survive and multiply in a variety of environments with different availability of nutrients. The pathogen possesses transport systems for various sugars allowing adaption to changing environmental conditions.

The genome of *L. monocytogenes* contains a significant number of ABC transporters phosphoenolpyruvate: sugar phosphotransferase (PTS) systems, most of which have not yet been fully characterized (Stoll and Goebel, 2010; Deutscher et al., 2014). Additionally, *L. monocytogenes* can utilize many carbon sources to produce biofilms on food processing surfaces (Tapia et al., 2020). In laboratory conditions, *L. monocytogenes* can grow in commonly used bacteriological media. Increased growth rates occur in the presence of fermentable sugars. However, nutritional requirements of *L. monocytogenes* are strain-dependent and reflect the ability of the pathogen to survive in a wide range of environmental conditions (Lungu et al., 2009). Premaratne et al. (1991) have shown that *L. monocytogenes* requires glucose, glutamine, leucine, isoleucine, arginine, methionine, valine, cysteine, riboflavin, biotin, and thiamine for growth. On the other hand, fructose, mannose, cellobiose, trehalose, maltose, glycerol, glucosamine, and N-acetylglucosamine support its growth in the absence of glucose. Tapia et al. (2020) have observed that the utilization of lactose by *L. monocytogenes* triggers a strong σ^B^-dependent stress response, which may have implications for *L. monocytogenes* in the food chain. Tapia et al. (2020) have demonstrated the potential role of lactose in the activation of the σ^B^-dependent stress response, which may have implications for *L. monocytogenes* in the food chain. They have observed that the pathogen in the presence of lactose, compared to other carbon sources tested, increased virulence, biofilm production potential, and resistance to heat and acids (Tapia et al., 2020). Thus, *L. monocytogenes* ability to utilize lactose may increase its survival in food processing (dairy), resulting in a higher risk of reinfection. Collectively, the ability to use alternative energy sources, e.g., carbohydrate polymers, proteins, nucleic acids, and lipids, determines the survival and growth of the pathogen in the human gastrointestinal tract (Lungu et al., 2009).

Zhou et al. (2012) have found that limited access to nutrients increased the biofilm formation ability of *L. monocytogenes*. Also, the presence of food by-products (meat juice, pork serum, or fat) in the processing environment stimulated biofilm formation (Van Houdt and Michiels, 2010). *Listeria monocytogenes* in the biofilm are resistant to disinfection methods. Therefore, efficient cleaning and disinfection procedures are of great importance.

### Reactive Oxygen Species

Disinfectants, commonly used in the food industry, trigger in bacteria oxidative stress. Reactive oxygen species (ROS), such as hydrogen peroxide or hydroxyl radicals, can be produced as by-products of metabolism or during reduction of the respiratory chain or other stress factors (Bowman et al., 2010; Burgess et al., 2016). In order to withstand exposure to oxidizing agents, e.g., sodium hypochlorite, cells must activate mechanisms to repair damages of proteins, the cytoplasmic membrane, and nucleic acids. *Listeria monocytogenes* under oxidative stress activates a survival strategy that includes the expression of *sigB*, cold and heat shock proteins, proteases (ClpC, ClpP, and GroEL), and ROS detoxification systems (Manso et al., 2020). ROS detoxification systems include superoxide dismutase (Sod), catalase (Kat), and alkylhydroperoxidase (AhpCF; Bucur et al., 2018). Strains without Kat and Sod activity showed increased sensitivity to oxidative stress and were low virulent (Archambaud et al., 2006; Azizoglu and Kathariou, 2010). Also, ferritin plays an essential role in the oxidative stress response of *L. monocytogenes* (Dussurget et al., 2005). Rea et al. (2005) have reported that *L. monocytogenes* ∆perR mutants (peroxide regulon repressor) displayed elevated sensitivity to hydrogen peroxide and limited growth.

Manso et al. (2020) have shown that oxidative stress is temperature-dependent. The lower temperature increased oxidative stress response. Both stressful conditions caused similar damage to nucleic acids and cell membranes (Manso et al., 2020). Most of the disinfectants used in the food industry are applied at low temperatures, favoring the acquisition of resistance by *L. monocytogenes*. Therefore, food manufacturers should consider the type of active substances in disinfectants and the disinfection process temperature.

### Disinfectants

One method to maintain food and consumer safety is the use of appropriate cleaning and disinfection procedures. Chemical preparations are most common. An important problem is the acquisition of resistance to disinfectants by *L. monocytogenes* due to the residues of chemical substances in sub-lethal concentrations after disinfection. The most commonly used disinfectants for *L. monocytogenes* are quaternary ammonium compounds (QACs) and chlorine-based biocides (Minarovicova et al., 2018; Aryal and Muriana, 2019). *Listeria monocytogenes* strains resistant to disinfectants contribute to sporadic cases as well as outbreaks of listeriosis. The antimicrobial effectiveness of biocides may be affected by the presence of organic pollutants. Also, the biofilm structure protects *L. monocytogenes* against the action of disinfectants (Duze et al., 2021). The main cause of resistance of *L. monocytogenes* to antimicrobial agents is horizontal gene transfer (HGT) of mobile genetic elements such as plasmids and transposons carrying resistant genes. Another mechanism relies on the activation of efflux pump systems. To date, six genes (*mdrL*, *lde*, cassette *bcrABC*, *qacH*, *emrE*, and *emrC*), located on mobile genetic elements, related to the efflux system, have been identified in *L. monocytogenes* in response to disinfectants action. Table 3 presents genes conferring biocide tolerance. Two major efflux pump genes, *mdrL* and *lde*, are present in nearly all *L. monocytogenes* serotypes (Haubert et al., 2019). The MDRL pump detoxifies macrolide, cefotaxime, heavy metals, and ethidium bromide (EtBr). In turn, the Lde pump detoxifies fluoroquinolones, antibiotics, and intercalating dyes such as EtBr and acridine orange (Mata et al., 2000). Jiang et al. (2019) confirmed the role of MdrL in disinfectants tolerance in *L. monocytogenes* (in the survival of biocidal stress in food). Other efflux pump genes responsible for the increased tolerance to QAC are *emrE* and *emrC* genes, located on the LGI1 genomic mobile island and pLMST6 plasmid, respectively (Kovacevic et al., 2016; Kremer et al., 2017). Kropac et al. (2019) showed that plasmid pLMST6 increased tolerance only to QAC-based disinfectants. However, as plasmids aid *L. monocytogenes* to survive in FPE, they may help in biocide tolerance. Kremer et al. (2017) have shown a link between isolates with plasmid pLMST6, pump efflux, *emrC* gene, and reduced antibiotic susceptibility. In turn, Noll et al. (2020) have studied the impact of adaptation *L. monocytogenes* against antimicrobials. *Listeria monocytogenes* cells after exposure to benzalkonium chloride showed a 2-fold increase in MIC compared to the disinfectant treatment and reduced sensitivity to antibiotics (ceftriaxone, gentamicin, linezolid, tetracycline, and a combination of trimethoprim and sulfamethoxazole). The tolerance to disinfectants does not always induce cross-resistance to antibiotics. Roedel et al. (2019) have revealed that tolerance to biocides did not induce AMR. Antibiotic resistance in *L. monocytogenes* may severely influence public health due to poor treatment outcomes and sequelae treatment failure. The frequent use of disinfectants in the food processing industry can lead to selective pressure and promote the absorption of plasmides, e.g., containing brcABC from other *L. monocytogenes* strains (Hingston et al., 2019).

**Table 3:** Genes involved in biocide tolerance in *L. monocytogenes*.

The source of the isolation *L. monocytogenes*	Biocide and exposure time	Presence/expression of genes	Reference
*L. monocytogenes* 08–5,578 [clinical strain responsible for the Canadian deli meat listeriosis outbreak (2008)]	Benzalkonium chloride – 16 h	*emrELm*	Kovacevic et al., 2016
Isolates from product contact and non-product contact surfaces	Benzalkonium chloride – 24 h	Transposon *Tn6188*	Ortiz et al., 2016
*L. monocytogenes* H7550 (strain implicated in the 1998–1999 multistate outbreak involving contaminated hot dogs)	Benzalkonium chloride – 48 h	*bcrABC*	Elhanafi et al., 2010
Isolates from nine Norwegian meat- and salmon processing plants	Benzalkonium chloride – 48 h	*bcrABC*	Møretrø et al., 2017
*L. monocytogenes* strains from Switzerland and Finland	Benzalkonium chloride – 48 h	*bcrABC, emrE*	Meier et al., 2017
Isolates from food and food processing plants	Benzalkonium chloride – 7 days	*mdrL*	Yu et al., 2018
Isolates from foods and food processing plants	Benzalkonium chloride, Cadmium & arsenic – 48 h	*CadA 1, CadA2*	Ratani et al., 2012
Isolates from food and food-processing environments in southern Brazil	Benzalkonium chloride & Cadmium chloride – 48 h	*mdrL, lde*	Haubert et al., 2019
Isolates from a dairy cattle farm	Benzalkonium chloride, Virkon, H_2_O_2_, and sodium Hypochlorite – 12-48 h	*qacED1*	Mohammed et al., 2017
Isolates from food production facilities	Benzalkonium chloride and Cetylpyridinium chloride – 72 h	*Pdu*, *Cob-cbi*, and *Eut*	Fox et al., 2011

It is relevant to control the used disinfectants to ensure the effective elimination of microorganisms from the food industry. In our opinion, periodical change of the active substances would have a beneficial impact. Such an action would limit the acquisition of resistance traits among microorganisms. Food manufacturers should monitor the effectiveness of agents and avoid extensive storage of open packages of disinfectants.

### Bacteriocins

Researchers have been constantly searching for new methods allowing microbes elimination from the FPE. These methods should be cheap, environmentally friendly, and, above all, safe for consumers. One alternative is the use of bacteriocins. Bacteriocins are antimicrobial peptides produced extensively by lactic acid bacteria. These molecules are mainly active against Gram-positive bacteria, including *L. monocytogenes* (Chikindas et al., 2018). Bacteriocins are natural and safe food preservatives (Silva et al., 2018). Nisin is the class I bacteriocin, approved as a food preservative. *Listeria monocytogenes* resistance to nisin is associated with changes in the cytoplasmic membrane. Ming and Daeschel (1993) have demonstrated an increased proportion of straight-chain fatty acids and a decreased branched-chain fatty acids ratio. In turn, Wu et al. (2018) have reported that *L. monocytogenes* bacilli exposed to a high nisin concentration had lowered expression of genes *dltA* and *dltB*, indicating that D-alanine residue is not linked to bacteriocin resistance. Scientists emphasized the role of regulation of the expression of two cell wall synthesis-associated genes: *lmo2714* (membrane-anchored peptidoglycan protein) and *lmo2522* (for cell wall-binding protein with possible effects on nisin tolerance; Wu et al., 2018). Other genes/operons that affect nisin resistance include *lmo2229* (Collins et al., 2012), *telA* (Collins et al., 2010a), *mprF* (Thedieck et al., 2006), *anrAB* (Collins et al., 2010b), and *dltABCD* (Abachin et al., 2002). Many class II bacteriocins with antilisterial activity, including pediocin, sacacin P, leukocin, enterococin, creaticine Y105, garvicin, and linocin M18 have been also isolated and characterized (Ríos Colombo et al., 2018). Natural resistance of *L. monocytogenes* ranges from 1 to 8%, depending on bacteriocin and tested strain (Macwana and Muriana, 2012).

*Listeria monocytogenes* response to bacteriocins involves different mechanisms, i.e., changes in cell structures and the participation of relevant proteins which work together.

## Stress Adaptation

*Listeria monocytogenes* can regulate and fine-tune gene expression to adapt to diverse stress conditions encountered during foodborne transmission (Orsi et al., 2021). The mechanism allowing *L. monocytogenes* to respond to stress conditions is the cross-talk between σ^B^ and PrfA.

Application of sublethal doses of stress factors in the FPE may increase AMR or stimulate the resistance to other stress conditions (cross-resistance) and higher doses of the same stress (stress adaptation; Faezi-Ghasemi and Kazemi 2015). Two-component signal transduction systems (TCS) enable bacteria to detect and adapt to a variety of stressors. The prototype modular TCS consists of a periplasmic sensor histidine kinase (HK) and a cognate cytoplasmic response regulator (RR; Gao and Stock, 2009). The action of TCS relies on the phosphorylation of proteins. In a direct phosphotransfer system, the HK sensor domain detects the stimulus leading to ATP-dependent autophosphorylation at a specific histidine (His) residue catalyzed by the kinase domain. Cognate RR then catalyzes the transfer of a phosphoryl group to its own aspartate (Asp) residue in the regulatory domain. Phosphorylation of the RR regulatory domain leads to activation of the effector domain and ultimately to an appropriate response to a specific stimulus (Casino et al., 2010).

Mobile genetic elements play a crucial role in the environmental adaptation and stability of *L. monocytogenes*. Such elements are common among *L. monocytogenes* isolates from FPEs (Hurley et al., 2019) and may contain genes mediating tolerance to heat shock (Pöntinen et al., 2017), salt and acid stress (Hingston et al., 2019; Naditz et al., 2019), and biocides (Meier et al., 2017). Also, plasmids are relevant for rapid adaptation to changing environmental conditions. The frequency of plasmids in *L. monocytogenes* is up to 79% (Elhanafi et al., 2010). The majority of data concerns plasmids containing genes conditioning resistance to disinfectants (Elhanafi et al., 2010; Rakic-Martinez et al., 2011), heavy metals (Rakic-Martinez et al., 2011), increased acid tolerance, and sensitivity to cold (Hingston et al., 2017), reduced tolerance to salt stress (Zhang et al., 2018), and antibiotics (Hadorn et al., 1993). Plasmids were more frequently isolated from food strains than from clinical strains (McLauchlin et al., 1997; Hingston et al., 2019). More, plasmids were more common among recurrent *L. monocytogenes* strains (75%) than among sporadic strains (35%) isolated from food or food processing plants (Harvey and Gilmour, 2001). These observations suggest that genes found in *L. monocytogenes* plasmids may be helpful for the pathogen’s survival in such environments (Hingston et al., 2019). A phenomenon found in the food industry is also HGT. The phenomenon is responsible for features critical to the evolution, such as antibiotic resistance or increased virulence. HGT ensures increased survival of *L. monocytogenes* strains exposed to stress factors related to FPE, which prevents their elimination, especially in places that are difficult to clean (Palma et al., 2020).

## Cross-Resistance

Cross-resistance is an essential aspect in the food industry, which often applies many stressors to assure food safety and food quality (Bergholz et al., 2012). Bergholz et al. (2012) have found that pre-exposure to osmotic stress enabled bacterial growth at low temperatures, whereas cold shock-induced cross-protection against salt stress. More, *L. monocytogenes* subjected to acidic conditions displayed increased resistance to H_2_O_2_, heat shock, ethanol, and oxidative and osmotic stress (Lou and Yousef, 1997; Ferreira et al., 2003). Adaptation to acid conditions may also increase *L. monocytogenes* resistance to nisin. Since the sublethal concentration of acids is frequently applied during food processing, cross-resistance of the acid-adapted cell is of great importance (Mastronicolis et al., 2010). Alonso-Hermando et al. (2009) have revealed that sodium hypochlorite induced AMR of *L. monocytogenes*. In turn, Faezi-Ghasemi and Kazemi (2015) have demonstrated increased susceptibility of *L. monocytogenes* to penicillin, ampicillin, gentamycin, tetracycline, trimethoprim/sulfamethoxazol, and rifampicin after stress exposure [7% NaCl, pH = 5.0, and ethanol (5% w/v)]. The researchers claimed that an acid environment leads to an influx of protons and anions to the cell cytoplasm, disturbing its metabolic functions and inducing the damage of proteins, nucleic acids, and cell membranes (Faezi-Ghasemi and Kazemi 2015). The knowledge of the cross-resistance phenomenon may help in the appropriate designing of food processes, limiting the risk for public health.

## Conclusion

In the presented review, we summarize the current knowledge about the adaptation of *L. monocytogenes* to stress factors in food processing plants. Although we know a lot about the stress response of *L. monocytogenes*, the pathogen is still present in the FPE and is a threat to the consumer. Mobile genetic elements determine the acquisition of new resistance traits to physical and chemical disinfection methods among *L. monocytogenes* strains. Food producers should not underestimate the risk of microorganisms present in the processing environment, especially *L. monocytogenes*. Therefore, high sanitary regimes and food processing control are indispensable for the reduction of foodborne listeriosis. Understanding the mechanisms of adaptation to environmental stress would significantly develop new, efficient, and cost-effective methods of controlling *L. monocytogenes* in the food industry. The knowledge of *L. monocytogenes* biology and constant control of the pathogen in FPE are critical to ensure food production safety.

## Author Contributions

NW-K and KS: conceptualization and project administration. KG-B and EW-Z: formal analysis. NW-K and JK: writing – original draft preparation and visualization. KS, KG-B, and EW-Z: writing – review and editing. KS and EG-K: supervision. All authors contributed to the article and approved the submitted version.

## Conflict of Interest

The authors declare that the research was conducted in the absence of any commercial or financial relationships that could be construed as a potential conflict of interest.

## Publisher’s Note

All claims expressed in this article are solely those of the authors and do not necessarily represent those of their affiliated organizations, or those of the publisher, the editors and the reviewers. Any product that may be evaluated in this article, or claim that may be made by its manufacturer, is not guaranteed or endorsed by the publisher.
